# Enhanced Production of Furfural via Methanolysis of Wood Biomass with HCl Gas

**DOI:** 10.1002/cssc.202401291

**Published:** 2024-11-06

**Authors:** A. Topias Kilpinen, Neptun Yousefi, Eero Kontturi

**Affiliations:** ^1^ Department of Bioproducts and Biosystems Aalto University P.O. Box 16300 Aalto FI-00076 Finland

**Keywords:** Alcoholysis, Biomass conversion, Hemicellulose degradation, Platform chemicals

## Abstract

This study explores the production of furfural, xylose and methylxylosides through the methanolysis of wood flour using anhydrous HCl gas. The process involves methanolysis of wood flour with HCl gas under pressure to generate methylxylosides, which are subsequently converted to xylose and furfural via autohydrolysis in a Parr batch reactor system. The methanolysis was conducted in temperature‐controlled HCl gas reactor employing 24 h reaction time and 50 % methanol content in wood flour. During the methanolysis step with HCl gas, 65 % of the available xylan in wood flour was converted to water‐soluble methylxylosides, xylose, xylooligosaccharides (XO) and water‐soluble methyl xylooligosaccharides (MXO). Methanolysis filtrates were then autohydrolyzed with Parr 50 mL batch reactor system to xylose and furfural in two different pH values at 180 °C. The highest furfural yield of 91 % from methanolysis filtrate was achieved with pH 1.2 and 25 min reaction time.

## Graphical abstract

Anhydrous HCl gas was employed to catalyze the methanolysis of aspen wood flour in a gas‐solid system to produce methyl xylosides. These methyl xylosides were then separated and hydrolyzed to furfural in a water environment with the Parr reactor system. Furfural yield during the hydrolysis step was 91 %, while the overall yield of furfural from aspen was 57 %.

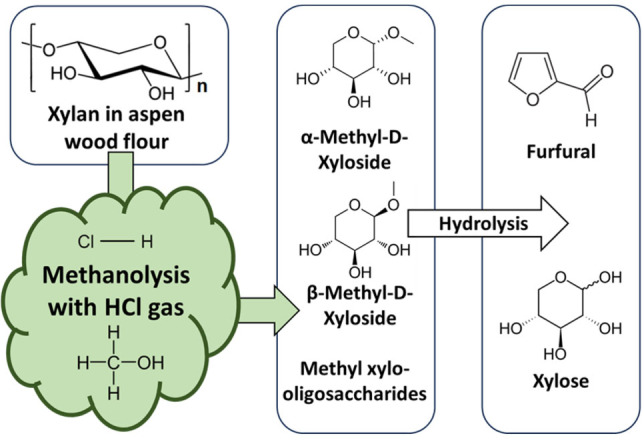

## Introduction

Due to shrinking fossil reserves and pressing environmental concerns such as global warming and air pollution, harnessing lignocellulosic biomass resources to produce fuels and chemicals sustainably has become crucial.^[1]^ Integral processing step to harnessing lignocellulosic biomass involves the breakdown of polysaccharides found in biomass, such as cellulose and hemicellulose, into monosaccharides like glucose and xylose. These monosaccharides can then undergo further processing to yield a diverse array of valuable products, including biofuels, solvents, organic acids, and platform chemicals.^[2]^ One example of possible platform chemicals produced from monosaccharides is furans. Furans, such as furfural and hydroxymethyl furfural (HMF), are formed from glucose and xylose via a series of acid‐catalyzed dehydration reactions. They are highly reactive compounds due to aldehyde groups and conjugated double bounds in their structure,^[3]^ and for this reason, they have gained attention as promising renewable platform chemicals for various industrial applications. For example, furfural serves as a precursor to chemicals such as furfuryl alcohol, tetrahydrofuran, levulinic acid and γ‐valerolactone, whereas HMF is a crucial intermediate for synthesizing pharmaceuticals, plastics and biofuels.^[4]^

In furfural production, pentosan sugars in hemicellulose are first hydrolyzed into xylose, which is then dehydrated to form furfural. While the initial hydrolysis step is fast and efficient, the dehydration step faces challenges. During the dehydration step, furfural and xylose‐furfural intermediates are prone to undergo complex side reactions such as resinification, condensation cross‐polymerization, and fragmentation reactions, resulting in low furfural yield. Process‐wise, furfural can be produced via a one‐ or two‐step process. The one‐step process entails the hydrolysis of pentosans to xylose and a concurrent dehydration to furfural. The two‐step process involves the hydrolysis of hemicellulose under mild conditions and subsequent dehydration of xylose into furfural.^[6]^ The production of HMF proceeds in a similar manner, but with hexosans as a starting material.

This study proposes a new two‐step pathway to produce furfural from aspen wood flour. In the first processing step, xylan in aspen wood flour is converted to methyl xylosides and methyl xylooligosaccharides (MXO) via HCl gas catalyzed methanolysis. In the second processing step, methyl xylosides and MXOs from the first step are converted to furfural during autohydrolysis in water environment.

**Scheme 1:**
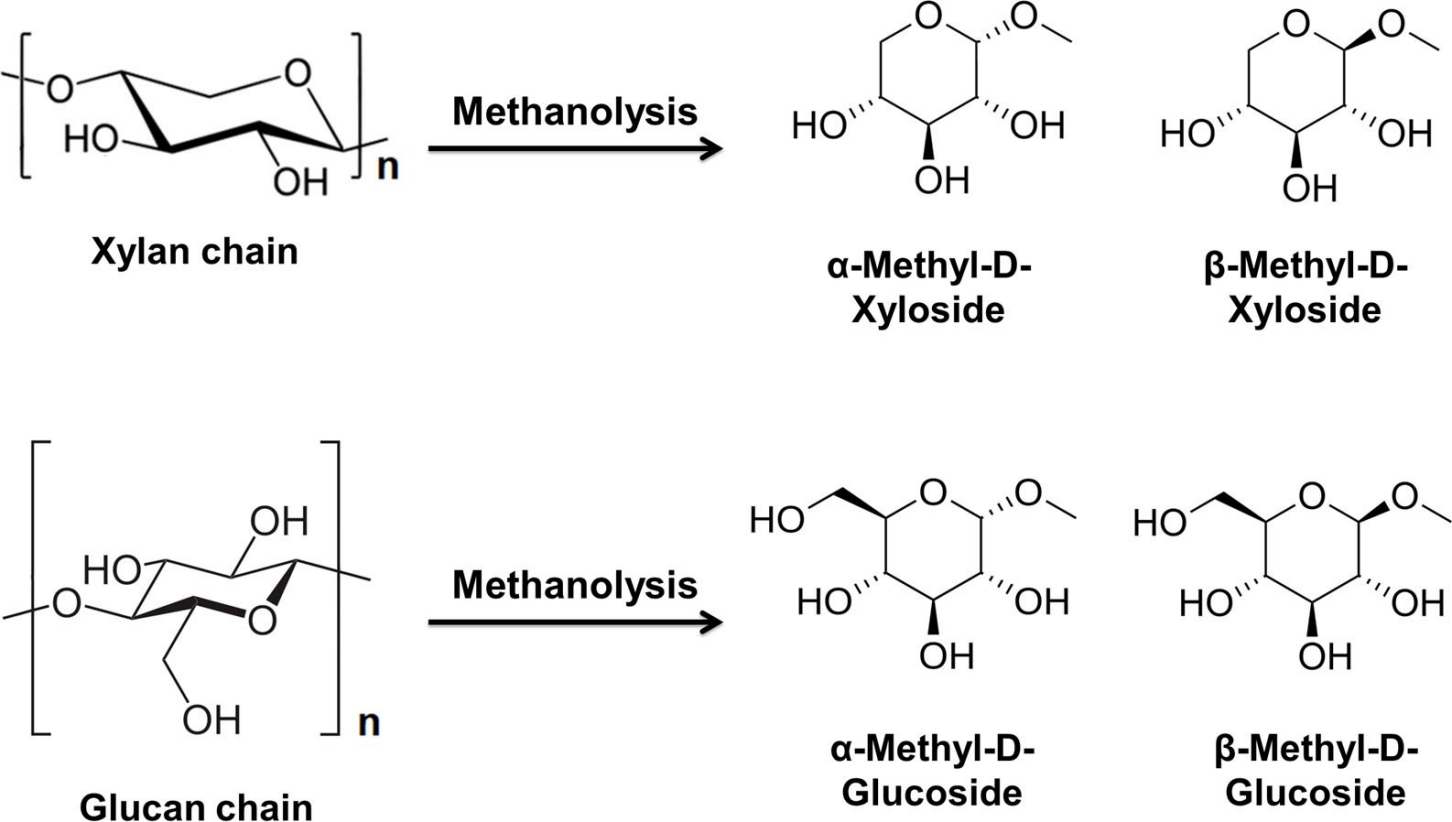
Methyl glycosides formed from xylan and glucan during methanolysis.

Methanolysis is a chemical process in which lignocellulosic biomass undergoes a reaction with methanol in the presence of an acid catalyst. During methanolysis, the hydroxyl group in methanol functions as a nucleophile, cleaving the glycosidic bonds of (hemi)cellulose and the acid‐ester bonds of lignin compounds in the presence of an acid catalyst. This interaction leads to the depolymerization of aromatics and polysaccharides within lignocellulose.^[7]^ During methanolysis, cellulose and hemicellulose are depolymerized into methyl glycosides and monosaccharides. Methyl glycosides are compounds formed by the substitution of a hydroxyl group in a glycoside molecule, for example glucose or xylose, with a methyl group (Scheme 1). These methyl glycosides can then be further processed to obtain the desired chemicals or biofuels such as furfural, alkyl levulinates and alkyl furans.[^7^, ^8^]

Methanolysis has several advantages for biomass fractionation when compared to more commonly utilized acid catalyzed hydrolysis. Firstly, methanol can be recovered through simple distillation, thereby reducing both the overall energy consumption and separation costs associated with the process. Secondly, alcohol‐derived decomposition products, such as hydrogen and alkoxy moieties, can suppress the formation of certain intermediates during methanolysis.^[7]^ This intermediate suppression results in the lower formation of unwanted side products, such as discoloring humins, during the process.^[9]^ Thirdly, decomposed non‐polar products from biomass have a higher solubility than methanol due to the lower dielectric constant when compared to water. This better dissolution efficiently prevents the re‐polymerization of the methanolized products.[^7^, ^10^] Previous studies of converting biomass with acid catalyzed methanolysis have mostly focused on liquid‐solid systems, employing higher temperatures (140–400 °C) and low acid concentrations to depolymerize lignin and hemicellulose to create bio‐oil.[^7^, ^10^, ^12^] However, no‐one has tried to catalyze the methanolysis of biomass with anhydrous HCl gas in a gas‐solid system. Anhydrous HCl gas has been employed in various studies to catalyze hydrolysis to produce monosaccharides, oligosaccharides, and cellulose nanocrystals (CNCs) from biomass with often superior outcome.^[14]^ Similarly to hydrolysis with HCl gas, methanol containing biomass is exposed to HCl gas under pressure in HCl (g) methanolysis. This leads to the protonation of methanol and catalyzes methanolysis *in situ* inside the biomass. The advantages of HCl gas catalyzed methanolysis are easy acid recovery via evaporation, lower required reaction temperature and lower amount of chemicals in circulation when compared to a liquid‐solid system.[^7^, ^20^] In addition, the suppression of intermediate product formation during methanolysis could decrease the formation of unwanted side products, such as humins, when compared to hydrolysis with water.^[11]^

Formed methyl xylosides and MXOs can be converted to furfural via autohydrolysis similarly to xylose and xylooligosaccharides (XO). As methyl xyloside is converted to furfural through a more complicated reaction pathway than xylose, this route should reduce the possibility of undesirable side reactions during dehydration, resulting in higher yields.^[21]^

The aim of this study is to present a novel two‐step process for the production of furfural from the biomass. In the first step aspen wood flour is subjected to HCl gas catalyzed methanolysis to produce methyl xylosides and MXOs. In the second step produced methyl xylosides and oligosaccharides are converted to furfural via autohydrolysis. Hydrolysis filtrates have been subjected to autohydrolysis in various studies to produce furans,^[24]^ but so far no‐one has subjected methyl xyloside and MXO containing methanolysis filtrates to autohydrolysis to produce furfural.

## Results and Discussion

### Carbohydrate Analysis and Mass Loss During Methanolysis

Table 1. presents the results of the carbohydrate analysis of the starting material (aspen without extractives) and the hydrolysis residue. As can be seen from the table, the average mass loss when washing the sample after methanolysis with HCl gas was roughly 30 % of the original mass. Glucan degradation was minimal and originates mostly from the non‐crystalline regions of cellulose. However, xylan degradation was significantly higher, and ca. 80 % of the available xylan was degraded during the methanolysis step. Degradation of Klason lignin was minimal during the methanolysis (1.4 %), but ca. 36 % of the acid soluble lignin (ASL) was degraded. A more in‐depth analysis of the effect of methanolysis on lignin will be conducted in future research.

**Table 1:** Carbohydrate analysis results of extracted wood flour and hydrolysis residue.

	Glucan‐%	Xylan‐%	Klason lignin‐%	ASL‐%	
Aspen (extractives removed)	49.1	17.3	20.3	2.6	
SD	1.6	0.6	0.1	0.2	
	Glucan loss‐%	Xylan loss‐%	Klason lignin loss‐%	ASL loss‐%	Mass loss‐%
Methanolysis residue	6.8	82.1	1.4	36.1	30.1
SD	1.7	0.8	2.3	2.8	2.7

### Methanolysis Results

**Figure 1:**
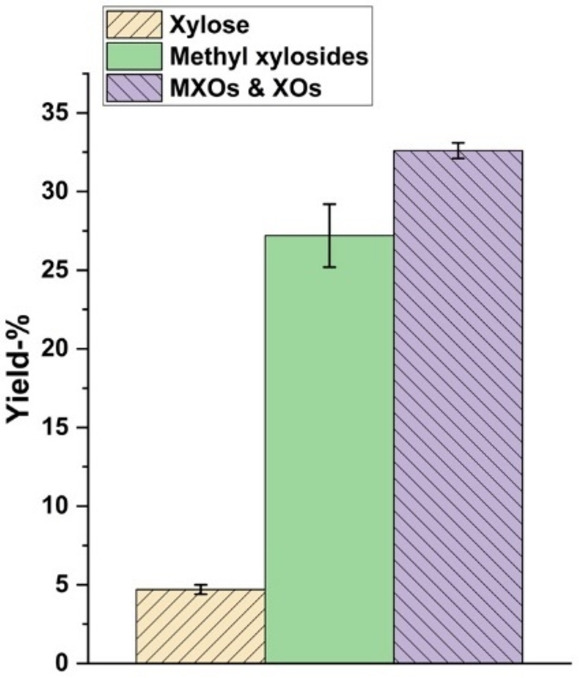
Yields of water‐soluble XOs, MXOs, xylose, and total methyl xylosides (α‐ and β‐ anomers) from methanolysis with HCl gas.

Figure 1 presents the calculated yields of water‐soluble XO, MXOs, xylose, and total methyl xylosides (α‐ and β‐ anomers). During methanolysis, the total conversion of available xylan to water‐soluble components was 65 %, and the conversion of available glucan was 4 %. Furfural, HMF and glucose amounts created during the methanolysis step were negligible. Numerical values for all components and yields from methanolysis are presented in SI Table S1. During the process of methanolysis using HCl gas, xylan was primarily converted into methyl xylosides (28 %), water‐soluble MXOs and XOs (32 %), and xylose (5 %). However, crystalline cellulose was not affected by methanolysis as only 4 % of the available glucans from aspen wood flour were converted to a water‐soluble form during methanolysis with HCl gas. When it comes to the generated methyl glycosides, only methyl xylosides can be accurately quantified with the available HPLC system due to the overlapping of methyl glucoside peaks with the xylose peak.

### Autohydrolysis Results

**Figure 2:**
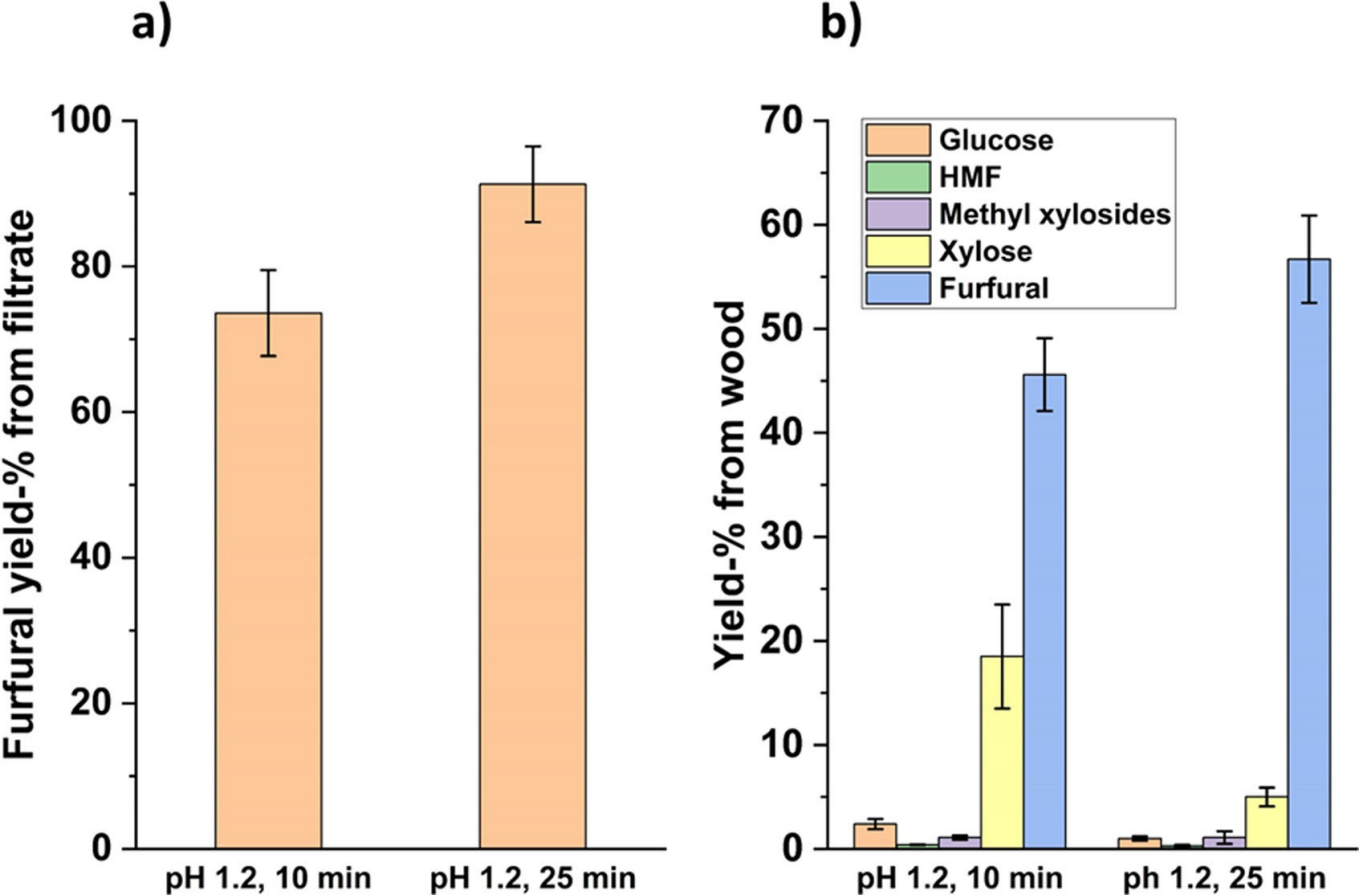
a) Furfural yields based on available xylose in methanolysis filtrate and b) yields from autohydrolyzed methanolysis filtrates based on available monosaccharides in methanolyzed wood flour.

The highest conversion of available xylose (methyl xylosides, xylose, MXOs, and XOs) in methanolysis filtrate to furfural was 91 % and was obtained using 25 min reaction time in pH 1.2 and 180 °C temperature (Figure 2a). This means that the overall furfural yield from available xylan from methanolyzed wood flour was 57 % in these reaction conditions (Figure 2b). In addition, when the xylose formation from methyl xylosides, MXOs and XOs is taken into account, we can notice that almost all of the available xylan in filtrates had been converted either to furfural or xylose during autohydrolysis. Numerical values for all components and yields from autohydrolysis are presented in SI Tables S2 and S3. No organic acids could be accurately quantified with HPLC due to unclear peaks or mixing with other components formed during methanolysis and autohydrolysis. Peaks for acetic acid and levulinic acid could not be detected, and the formic acid peak was obscured by an unknown peak (SI‐Figure S1).

### Comparison with HCl Gas Hydrolysis

Kilpinen et al. employed the same HCl gas reactor system for the production of water‐soluble carbohydrates from aspen wood flour. In that study, using similar conditions and HCl gas loadings for hydrolysis as in this one (24 h and 50 % dry matter content), but with water instead of methanol, no extractives removal and no vacuum evaporation, 85 % of glucan and 55 % of xylan were degraded.^[14]^ It is noteworthy that during methanolysis, mainly hemicellulose (xylan) degraded without much conversion to degradation products such as humins, contrary to HCl gas hydrolysis, which caused substantial humin generation. It is evident that under the applied temperatures and pressures employed, methanolysis with HCl gas is not able to break down crystalline cellulose.

### Comparison to Other Methanolysis Processes

Methanolysis results from this study were compared to some of the earlier studies that have employed more conventional methanolysis processes at high temperatures and dilute acid concentrations and are presented in Supporting Information Table S4.[^7^, ^11^, ^13^] When comparing the methanolysis with HCl gas to more conventional methanolysis processes employing dilute acids, three notable observations become apparent. Firstly, both processes lead to high xylan conversion, but the yield of methyl xylosides is higher with the HCl gas methanolysis process. This could be explained by the degradation of xylan and methyl xylosides in higher reaction temperatures employed during methanolysis with dilute acids. Secondly, the lignin yield from methanolysis with HCl gas is negligible when compared to lignin yields from dilute acid methanolysis. It appears that the lignin does not degrade in lower reaction temperatures employed in HCl gas methanolysis. Thirdly, the mass loss is lower with HCl gas methanolysis as mainly hemicellulose and non‐crystalline regions of cellulose are degraded. However, direct comparison is difficult due to variances in analytical methods and the way results are presented in the studies.

### Comparison of Furfural Yield with other Similar Studies Utilizing Autohydrolysis

**Figure 3:**
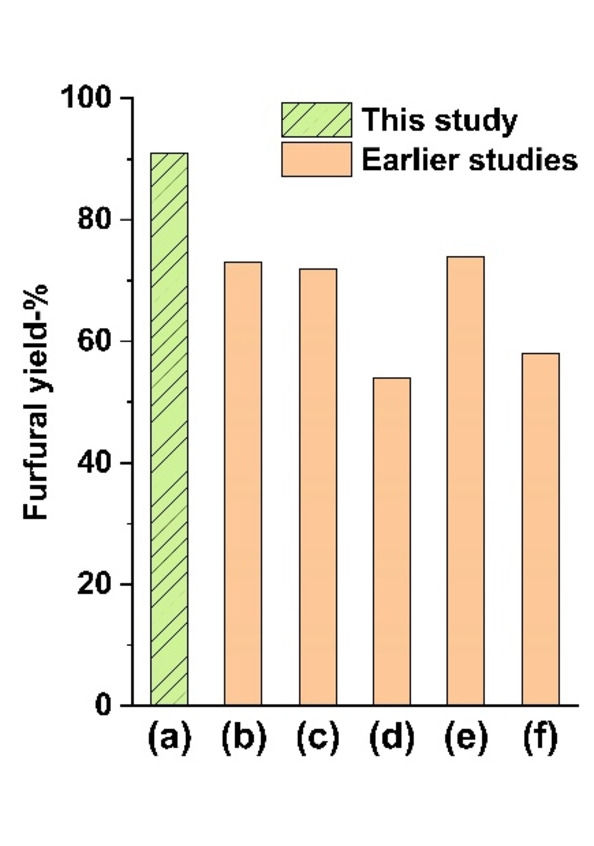
Comparison to previous furfural production studies. a) Autohydrolysis of HCl gas catalyzed methanolysis filtrates (this study), b) vapor‐releasing system,^[29]^ c) microwave assisted hydrolysis,^[25]^ d) two‐phase system,^[26]^ e) autoclaving with formic acid,^[27]^ and f) microwave assisted hydrolysis.^[28]^

Autohydrolysis results from this study were also compared to some of the earlier studies that utilized autohydrolysis to produce furfural. The furfural yields of these studies are presented in Figure 3. A more detailed comparison can be found in Supporting

**Scheme 2:**
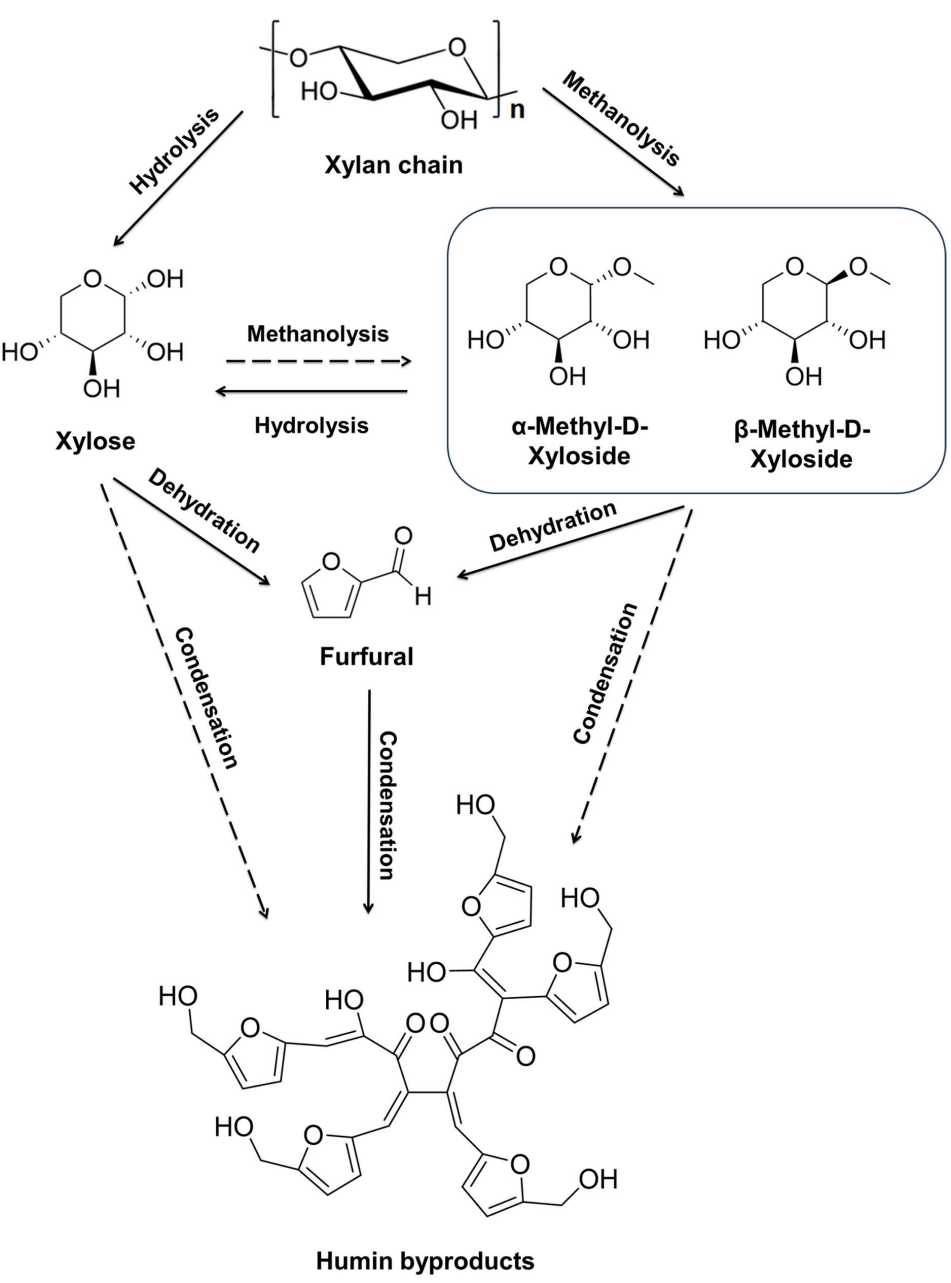
Simplified reaction pathways during methanolysis and autohydrolysis.

Information Table S5. As can be seen from Figure 3, the best furfural yields achieved in this study are higher (91 %) than in autohydrolysis processes employing a similar autohydrolysis of hydrolysis filtrates (54–74 %).[^25^, ^26^, ^27^, ^28^, ^29^] The higher furfural yield from methyl xyloside, when compared to xylose, can be attributed to its more complex reaction pathway and the protective effect of the methyl group/methanol against condensation reactions. The conversion pathway from methyl xyloside to furfural is more complicated, as methyl xyloside can either revert back to xylose, which subsequently transforms into furfural, or directly convert to furfural during hydrolysis. Simplified reaction pathways are illustrated in Scheme 2.^[21]^ In the direct route, methyl xyloside undergoes ring‐opening to form a chain methyl xyloside, followed by demethanolization to produce a five‐membered ring intermediate. Subsequently, two water molecules are successively eliminated to yield furfural. Additionally, methanol may play a protective role on the unsaturated carbonyl group of furfural, thereby impeding its polymerization into humins.^[22]^ Furthermore, the presence of the methyl group in methyl xyloside may hinder the acetal cyclization and condensation reactions necessary for humin formation.^[23]^

The main challenge with processes employing HCl gas has been the formation of sticky intermediate products when cellulose is degraded. However, it is noteworthy that during HCl gas‐catalyzed methanolysis, wood flour does not turn into a sticky intermediate product. This is because primarily the hemicellulose component is degraded during methanolysis. Similar behavior was observed in a study where HCl gas was used for the selective removal of C5‐sugars.^[20]^ Considering the relatively low required overpressure of 0.1 MPa, low reaction temperatures, and the ease of recovery for methanol and HCl after methanolysis, this process has significant potential for scaling up.

Scaling up of the HCl gas methanolysis process could be achieved by employing a modified screw extruder or a similar continuously operating mixing reactor made from acid‐resistant material. This kind of reactor could be employed for the pre‐hydrolysis of biomass to remove C5‐sugars prior to further processing. After methanol and HCl are removed via evaporation, the methyl xylosides and MXOs can then be extracted from the methanolysis residue with simple water washing. These extracted compounds can then be subjected to autohydrolysis, either in a batch or continuous autohydrolysis reactor, to produce furfural. Subsequently, the methanolysis residue can undergo further fractionation to produce glucose and lignin, enhancing the overall efficiency and yield of the process.

However, further research is still needed to optimize the methanolysis step to improve the overall MXO, XO and methyl xyloside yield from wood, as only 65 % of available xylan could be extracted from the wood flour during methanolysis. One promising research pathway would be to investigate the efficiency of HCl gas catalyzed methanolysis in overpressures above 0.1 MPa and with shorter reaction times. In addition, the effect of methanolysis on the cellulose and lignin components, as well as the potential formation of methyl chloride and dimethyl ether during methanolysis, needs to be investigated in further studies to assess the scalability of the process.

## Conclusions

Using temperature‐controlled HCl gas for methanolysis, it was possible to achieve a 28 % yield of methyl xyloside from aspen wood flour. The total xylan conversion to water‐soluble methyl xylosides, xylose, MXOs and XOs was 65 %. However, the employed reaction conditions were not effective in degrading crystalline cellulose or lignin. Additionally, the methanolysis filtrate containing methyl xylosides and methyl xylooligosaccharides could be converted to furfural with an 91 % yield during autohydrolysis, which is higher than in conventional autohydrolysis of xylan or xylose‐containing hydrolysis filtrates (54–74 %). The total furfural yield from methanolyzed raw material aspen was 57 %. These results suggest that combining methanolysis with subsequent autohydrolysis of methanolysis filtrates could offer a promising new pathway for producing furfural from biomass.

## Experimental Section

### Materials

Aspen (Populus Tremula) wood chips were provided by Avantium NV (the Netherlands). Aspen wood chips had a dry matter content of 99 %. Analytical grade furfural, 5‐hydroxymethylfurfural (HMF), acetic acid, formic acid, levulinic acid, 25 % analytical grade sulfuric acid, β‐methyl xyloside (≥99 % (GC), α‐methyl glucoside (99 %) and β‐methyl glucoside (≥99 % (HPLC and GC)) were purchased from Merck. Methanol, acetone and α‐D‐methyl xyloside (98 %) were purchased from VWR. We used Millipore‐grade water (resistivity 18.2 MΩ, conductivity 0.8 μS/cm) for chromatography analyses. Deionized water used in this work was obtained through a deionized water system equipped with a water softener, reverse osmosis, ion exchanger and UV‐light manufactured by Eurowater (conductivity <1 μS/cm).

### Methods

We determined the carbohydrate composition of oven‐dried acetone extracted aspen wood flour using the analytical method NREL/TP‐510‐42618.^[30]^ Sugars were quantified with high‐performance anion exchange chromatography with pulsed amperometric detection (HPAEC‐PAD) under the Dionex ICS‐5000 system (Sunnyvale, CA, USA). Millipore‐grade water was used as the mobile phase at a flow rate of 0.38 mL/min with a CarboPac PA20 column. Sugar composition in hydrolysis filtrates was determined according to the analytical method NREL/TP‐510‐42623.^[31]^ The dry matter content of the samples was determined according to the analytical method NREL/TP‐510‐42621.^[32]^ The concentrations of furans, organic acids, methyl xylosides and methyl glucosides were determined via high‐performance liquid chromatography (HPLC) by using Dionex UltiMate 3000 HPLC (Dionex, Sunnyvale, CA, USA) equipment outfitted with ultraviolet (UV) detector and Rezex ROA‐Organic Acid column (Phenomenex). Sulfuric acid solution (0.0025 mol/L) was used as the eluent at a 0.5 mL/min flow rate. The column temperature was 55 °C. Concentrations of furfural, HMF, acetic acid, formic acid, levulinic acid, α‐methyl glucoside, β‐methyl glucoside, α‐methyl xyloside and β‐methyl xyloside in the liquid samples were determined by the UV detector at wavelengths of 210 and 280 nm.

### Methanolysis with Anhydrous HCl Gas and Autohydrolysis of Filtrates

**Figure 4:**
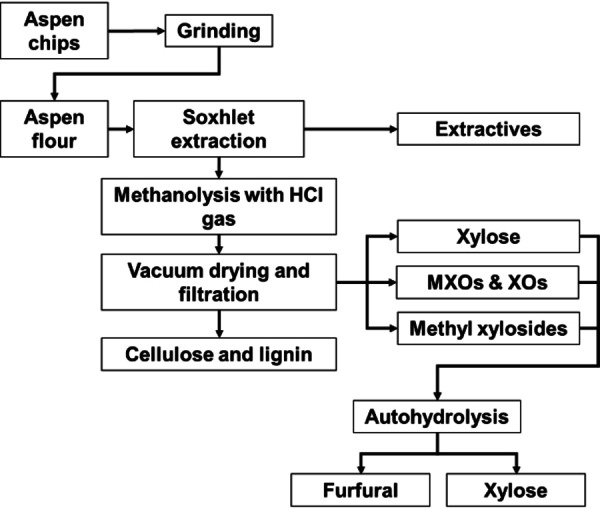
Process flow chart.

Figure 4 shows a flow chart of our process: pretreatment and methanolysis with HCl gas of aspen wood flour, followed by converting methyl xylosides to furans using autohydrolysis. Three separate methanolyses followed by autohydrolysis of methanolysis filtrate were conducted to ensure repeatability and to calculate mean values and standard deviation for yields. The aspen wood chips were ground to wood flour using Wiley mill M02 through a 1.9 mm screen. After the grinding step, the extractives were removed from aspen wood flour using a 6 h Soxhlet extraction with acetone. We dried the extracted wood flour in a 105 °C oven overnight on an open tray to remove residual moisture from the wood prior to methanol additon. Completness of drying was confirmed by a stable end weight. After oven drying, the wood flour was place to dessicator to prevent absorption of moisture from air during cooling. After the extracted wood flour had cooled down, we used 5 g of wood flour and added 5 g of methanol by spraying and mixing inside a large glass beaker to obtain 50 % dry matter content. The wood flour with 50 % methanol content was weighed and transferred to a 1 L Pyrex gas reactor bottle. Using the reactor bottle, we employed an HCl gas hydrolysis system for our methanolysis step.^[18]^ For the methanolysis, the reactor bottle containing 50 wt % wood—50 wt % methanol mixture was placed in an ice water bath (−1 °C) to cool down for 30 min. A cooling step is necessary to prevent excess heat formation during the HCl gas application phase. After the cooling step, 5 g of HCl gas was added to the reactor with occasional mixing of the flour by rotating the reactor bottle while tilted. Once 5 g of HCl had been added, the bottle was pressurized to 0.1 MPa overpressure with nitrogen. The bottle was kept in an ice bath during the HCl gas application step, and the time spent in the ice bath was 15 min. After 15 min, the ice bath was removed and the reactor bottle stayed in the fume hood at 21 °C, where the reaction bottle was kept for a total HCl gas reaction time of 24 h. For the last 10 min of HCl gas reaction time under pressure, the reaction bottle was placed inside a water bath (3.7 L, 55 °C) to speed up the methanolysis process. The cooling and heating configurations, as well as the change of color in aspen wood during methanolysis, can be found in Supporting Information (SI) in Figures S2 and S3. After the water bath, the overpressure from HCl gas and N_2_ was released through the reactors neutralization system (bubbling through 10 % NaOH solution) and the methanolized wood‐methanol mixture was subjected to vacuum evaporation in order to remove residual HCl and methanol. A photograph of the vacuum evaporation can be found in the SI (Figure S4). The evaporation was conducted by placing the reactor bottle into a water bath (100 °C, for 15 min) under 3 mbar vacuum from the water jet pump. Every 2 minutes of vacuum evaporation the air inlet valve was opened carefully to let air flow carry out evaporated components into water jet pump. During vacuum evaporation 98 % of methanol and residual HCl was removed from the methanolysis residue based on the weight loss. In the next step, 100 g of deionized water was added to the wood flour that had been treated with methanol and left to soak overnight. The following day, the samples were filtered through glass filters with a porosity of 4 (SI Figure S5). The methanolysis residue was washed with deionized water until reaching pH 5, then dried in a fume hood and weighed to determine the residual weight. The pH value of the filtrate was measured and analyzed for the monosaccharides (glucose and xylose) and total carbohydrate content using a DIONEX‐5000 HPLC system. For analyzing methyl glucosides, methyl xylosides, furfural, HMF, formic acid, acetic acid and levulinic acid in the filtrate, the Ultima 3000 HPLC system was employed. Further, we measured the composition of the starting material and methanolysis residue.

To produce furfural and xylose from methanolysis filtrates, 50 mL Parr reactor was employed. The photograph of the reactor system is presented in the SI (Figure S6). For the autohydrolysis methanolysis filtrates were hydrolyzed for 10‐ and 25‐minutes reaction time in 180 °C. The pH of the methanolysis filtrates was adjusted from 1.6 to1.2 with 1 M HCl (aq) prior to autohydrolysis. Reaction conditions were chosen based on earlier studies with similar pH range[^25^, ^28^] and on the results of preliminary tests with Parr‐reactor. To perform autohydrolysis, 30 mL of methanolysis filtrate was added to the reactor vessel. The mixture was then heated to 180 °C over a period of 11 min, and kept at 180 °C for 10 and 25 min. Finally, the mixture was cooled down to 30 °C over a period of 6 min by submerging the reactor vessel into a bucket of ice water. The sample was mixed at 300 rpm during autohydrolysis with the rotor inside the reactor vessel. The temperature profile during autohydrolysis is presented in the SI (Figure S7). Hydrolyzed filtrates were then analyzed using Dionex‐5000 for carbohydrates and Ultima 3000 HPLC system for methyl glucosides, methyl xylosides, furfural, HMF, formic acid, acetic acid and levulinic acid.

## Calculations

Yield calculations are based on the moles (N) of the products (HMF, Furfural, methyl xylosides) measured from filtrates and moles of available monosaccharides (arabinose, xylose, glucose) in methanolized aspen wood flour (Equation 1.

(1) $$Yield\;\%=\frac{N\left(product\right)}{\begin{array}{c} N(corresponding\;\\ available\;monosaccharides) \end{array}}\times 100$$

## Conflict of Interests

The authors declare no conflict of interest.

1

## Supporting information

As a service to our authors and readers, this journal provides supporting information supplied by the authors. Such materials are peer reviewed and may be re‐organized for online delivery, but are not copy‐edited or typeset. Technical support issues arising from supporting information (other than missing files) should be addressed to the authors.

Supporting Information

## Data Availability

The data that support the findings of this study are available from the corresponding author upon reasonable request.
