# Supplementary material for: Enhanced Production of Furfural via Methanolysis of Wood Biomass with HCl Gas
Source: ChemSusChem. 2024 Nov 6;18(3):e202401291. doi: 10.1002/cssc.202401291 (PMC11789968; doi:10.1002/cssc.202401291)
Supplement: Supplementary file 1 — Supporting Information [file CSSC-18-e202401291-s001.pdf]

ChemSusChem

Supporting Information

Enhanced Production of Furfural via Methanolysis of Wood Biomass with HCl Gas

A. Topias Kilpinen, Neptun Yousefi, and Eero Kontturi*

Supporting Information

Enhanced Production of Furfural via Methanolysis of Wood Biomass with HCl Gas

A. Topias Kilpinen [a], Neptun Yousefi [a] and Eero Kontturi [a]*
[a] Department of Bioproducts and Biosystems, Aalto University, P.O. Box 16300, FI-00076 Aalto, Finland
* Corresponding author: Eero Kontturi, eero.kontturi@aalto.fi

Table of contents

**Table S1.** Yields from methanolysis in numerical form	S2
**Table S2.** Furfural yields from autohydrolysis in numerical form based on available xylose in metholysis filtrate	S2
**Table S3.** Yields from autohydrolysis in numerical form based on available monosaccharides in methanolyzed wood flour	S2
**Figure S1.** Unknown peak overlapping the formic acid peak in HPLC	S2
**Table S4.** Comparison to earlier methanolysis studies	S3
**Table S5.** Comparison to previous furfural production studies	S4
**Figure S2.** Gas hydrolysis process	S5
**Figure S3.** Color change during methanolysis	S5
**Figure S4.** Vacuum evaporation setup	S6
**Figure S5.** Methanolysed sample after dilution and filtration	S6
**Figure S6.** Autohydrolysis equipment	S7
**Figure S7.** Temperature gradient with Parr reactor during 25 min reaction time	S7
**References**	S8

Number of Pages: 8

Number of Figures: 7

Number of Tables: 5

Table S1. Yields from methanolysis in numerical form.

	Xylose yield-%	Methylxylosides yield-%	MXO & XO yield-%	Water-soluble total glucan yield-%
Methanolysis	4.7	27.5	32.3	3.5
SD	0.3	2.5	0.6	0.3

Table S2. Furfural yield-% from available xylose in methanolysis filtrate.

	Furfural yield-%	SD
pH 1.2, 10 min	73.6	5.9
pH 1.2, 25 min	91.3	5.2

Table S3. Yields from autohydrolyzed methanolysis filtrates based on available monosaccharides in methanolyzed wood flour in numerical form.

	Glucose yield-%	HMF yield-%	Xylose yield-%	Furfural yield-%	Methylxyloside yield-%
pH 1.2, 10 min	2.4	0.4	18.5	45.6	1.1
SD	0.5	0.03	5.0	3.5	0.2
pH 1.2, 25 min	1.0	0.4	5.0	56.7	1.1
SD	0.2	0.1	0.9	4.2	0.6

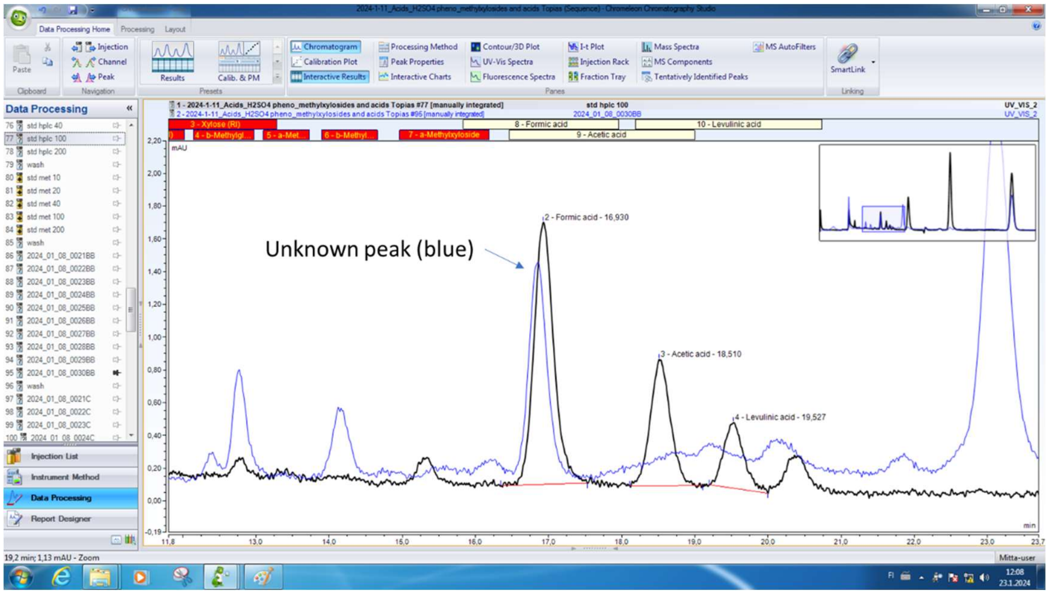

Figure S1. Unknown peak overlapping the formic acid peak in HPLC.

Table S4. Comparison to earlier methanolysis studies.

Process/ system	Conversion (wt-%)	Acid catalyst	Reaction time	Temperature (˚C)	Yield total methyl glycosides (%)	Yield Methyl xyloside (%)	Xylan conversion (%)	Yield Lignin (%)	Source
Methanolysis with HCl gas	33.7	HCl (gas)	24 h	-1 to 55	9.9	27.5	82.1	1.5	This study
High pressure reactor	52.25	H_2SO_4	30 min	160	24.85	12.32	85 (based on hemicellulose)	74	[1]
High pressure reactor	40.21	HCl (aq)	30 min	160	16.77	9.57	61.61 (based on hemicellulose)	50.25	[1]
100 mL PARR - autoclave	41.3	H_2SO_4 0.3 wt%	2 h	180	(12 wt-% from wood)	12	n.a.	86.4	[2]
Parr 4843 pressure reactor system	82.77	0.625% H_2SO_4	10 min	200	42.33 (total yield of sugars)	n.a.	n.a.	24.5	[3]

Table S5. Comparison to previous furfural production studies.

Process	Raw material	Acid concentration/pH	Reaction time	Temperature	Yield-% furfural	Source
Autohydrolysis of HCl gas catalyzed methanolysis filtrates	Methanolysis filtrate	pH 1.2	25 min	180˚C	91	This study
Vapor-releasing system	Biomass hydrolysate from autohydrolysis pretreatment	pH 3.5	3 h	200˚C	73	[4]
Microwave assisted hydrolysis	Flax shives	pH 1.12	20 min	180˚C	72.08	[5]
Two-phase system	Birch hydrolysis filtrate	n.a.	3 h	190˚C	54	[6]
Autoclaving with formic acid	Xylose	10g/L of formic acid	6.5 h (simultaneous distillation)	180˚C	74	[7]
Microwave assisted hydrolysis	Corn cob autohydrolysis filtrates	2% (v/v)	5 min	180˚C	37.06 (by weight, 58 by moles)	[8]

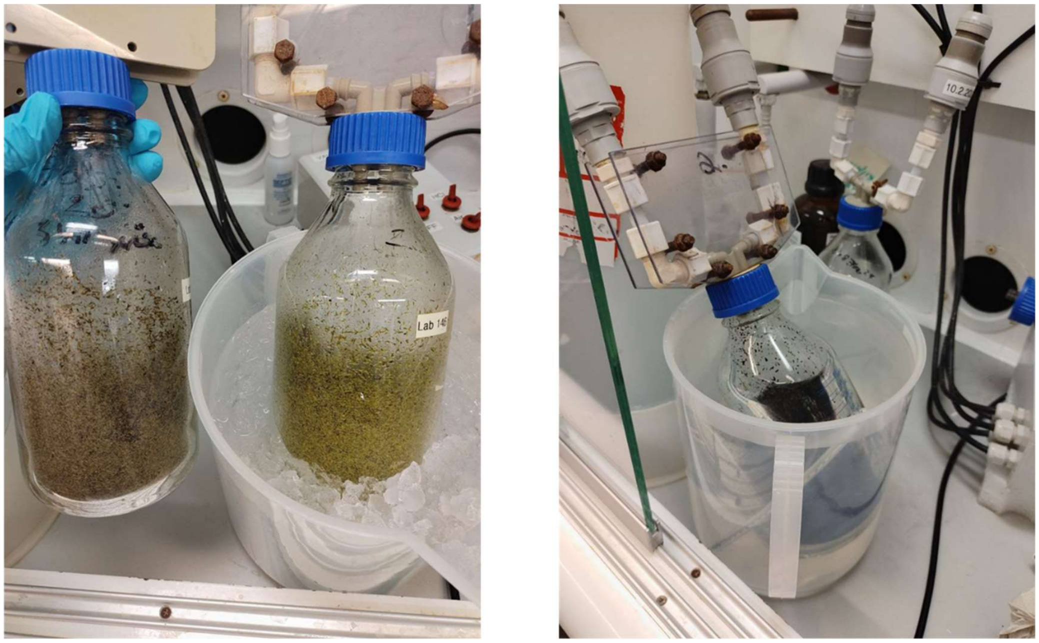

a) b)

Figure S2. Samples after and during 15 min gas application step under cooling in an ice bath (a). Sample in 55 °C water bath during the last 10 min of methanolysis (b).

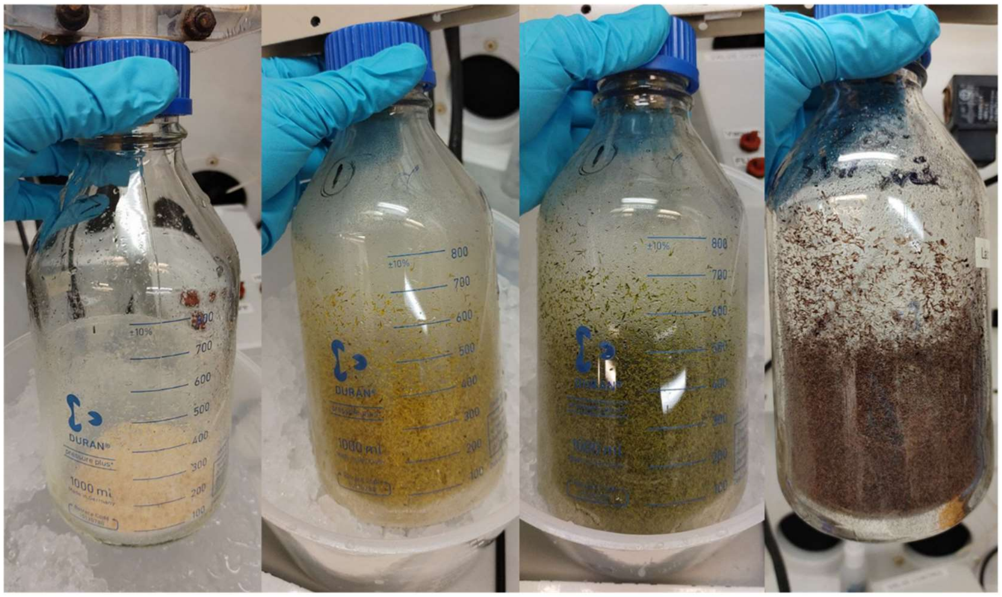

a) b) c) d)

Figure S3. Color change during 24 h methanolysis. Prior to HCl gas addition (a), after 5 min from HCl gas addition (b), after 15 min from HCl gas addition (c) and after 24 h from HCl gas addition (d).

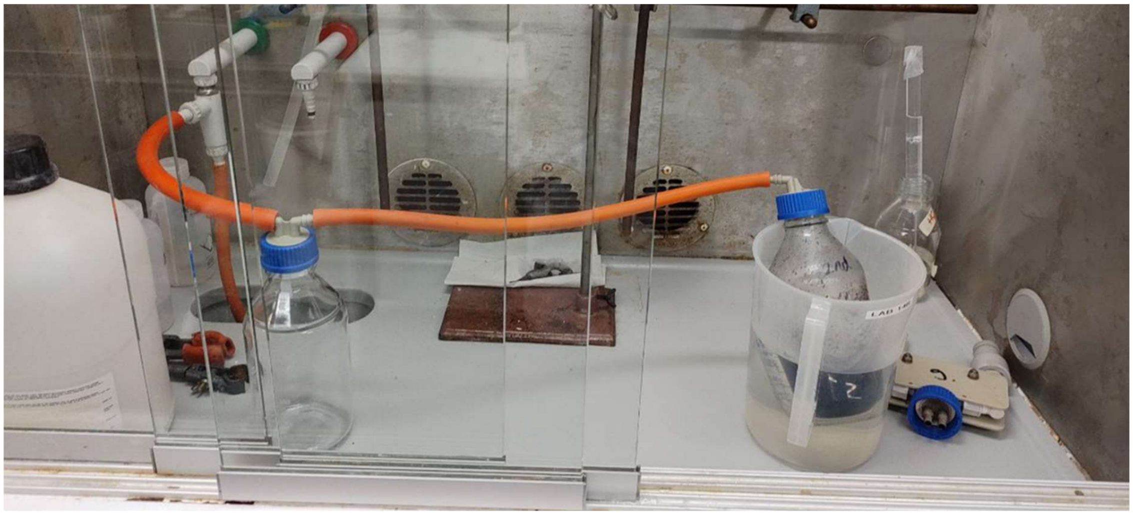

Figure S4. Vacuum evaporation system.

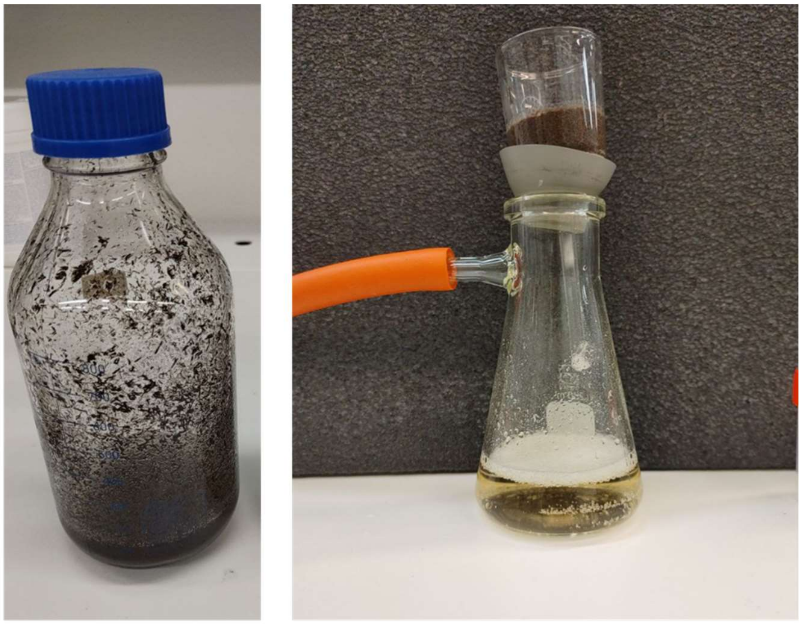

a) b)

Figure S5. Methanolyzed sample after water addition (a) and filtration of the sample through porosity 4 glass filter (b).

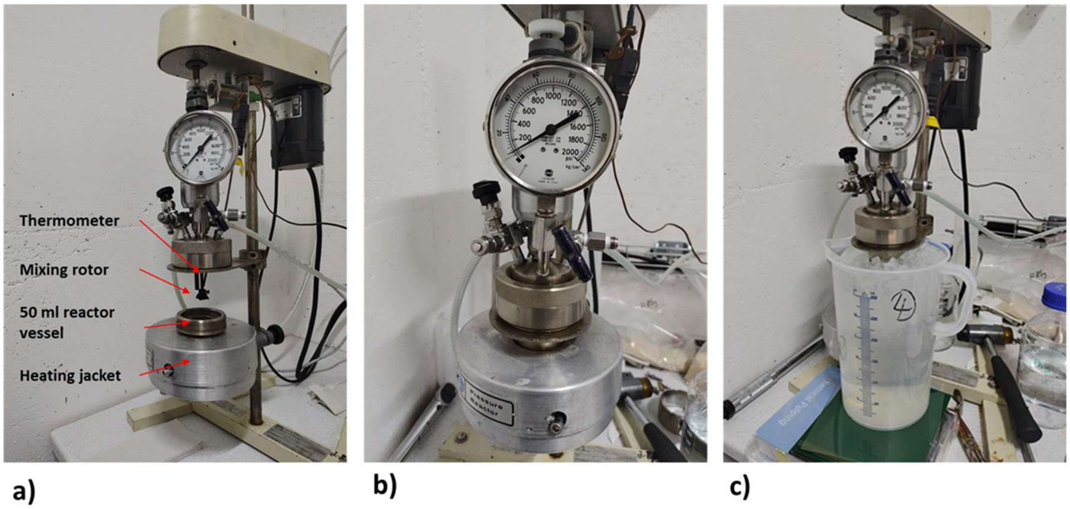

Figure S6. Reactor system employed for autohydrolysis (a), reactor system during heating phase (b) and reactor system during cooling phase (c).

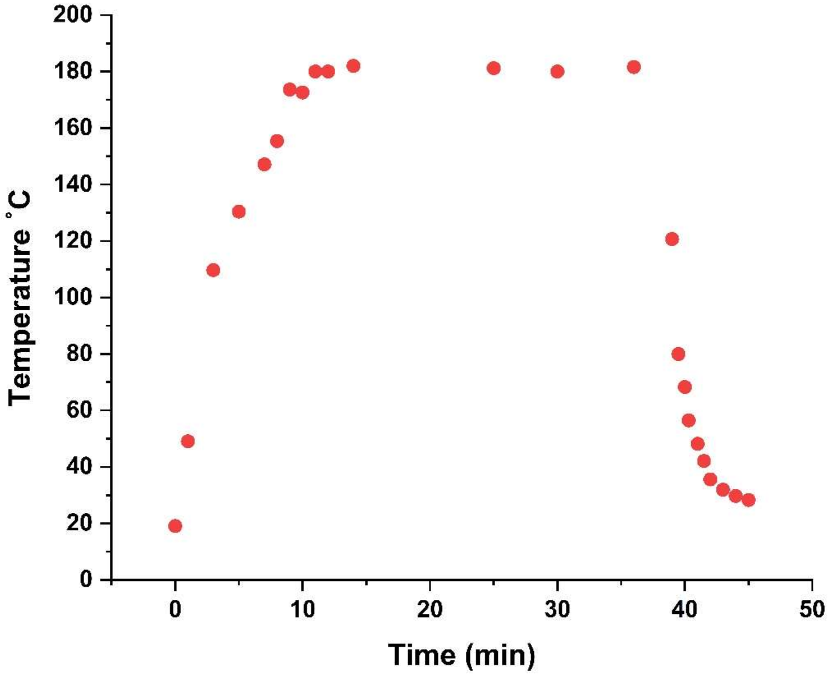

Figure S7. Temperature gradient with Parr reactor during 25 min reaction time.

References

[1] Q. Zhai, C.-y. Hse, F. Long, T. F. Shupe, F. Wang, J. Jiang, J. Xu, J. Agric. Food Chem. 2019, 67, 9840-9850.

[2] P. D. Kouris, X. Huang, X. Ouyang, D. J. van Osch, G. J. Cremers, M. D. Boot, E. J. Hensen, *Catalysts* **2021**, *11*, 750.

[3] J. Xu, X. Xie, J. Wang, J. Jiang, *Green Chem.* **2016**, *18*, 3124-3138.

[4] L. Liu, H.-m. Chang, H. Jameel, S. Park, *Bioresour. Technol.* **2018**, *252*, 165-171.

[5] O. Yemiş, G. Mazza, *Bioresour. Technol.* **2011**, *102*, 7371-7378.

[6] G. Gómez Millán, R. P. Bangalore Ashok, P. Oinas, J. Llorca, H. Sixta, *Biomass Convers. Biorefin.* **2021**, *11*, 2095-2106.

[7] W. Yang, P. Li, D. Bo, H. Chang, *Carbohydr. Res.* **2012**, *357*, 53-61.

[8] C. Sánchez, L. Serrano, M. A. Andres, J. Labidi, *Ind. Crops Prod.* **2013**, *42*, 513-519.
